# Engineering styrene biosynthesis: designing a functional *trans*-cinnamic acid decarboxylase in *Pseudomonas*

**DOI:** 10.1186/s12934-024-02341-0

**Published:** 2024-02-28

**Authors:** Ana García-Franco, Patricia Godoy, Estrella Duque, Juan L. Ramos

**Affiliations:** 1 Estación Experimental del Zaidín. Consejo Superior de Investigaciones Científicas, c/ Profesor Albareda 1, 18008 Granada, Spain; 2 Programa de Doctorado en Bioquímica y Biología Molecular, Universidad de Granada, Granada, Spain

**Keywords:** *Pseudomonas*, Aromatic hydrocarbons, Styrene, Synthetic genes, Decarboxylases, PAL enzymes

## Abstract

We are interested in converting second generation feedstocks into styrene, a valuable chemical compound, using the solvent-tolerant *Pseudomonas putida* DOT-T1E as a chassis. Styrene biosynthesis takes place from L-phenylalanine in two steps: firstly, L-phenylalanine is converted into *trans*-cinnamic acid (*t*CA) by PAL enzymes and secondly, a decarboxylase yields styrene. This study focuses on designing and synthesizing a functional *trans*-cinnamic acid decarboxylase in *Pseudomonas putida*. To achieve this, we utilized the “wholesale” method, involving deriving two consensus sequences from multi-alignments of homologous yeast ferulate decarboxylase FDC1 sequences with > 60% and > 50% identity, respectively. These consensus sequences were used to design *Pseudomonas* codon-optimized genes named *psc1* and *psd1* and assays were conducted to test the activity in *P. putida*. Our results show that the PSC1 enzyme effectively decarboxylates *t*CA into styrene, whilst the PSD1 enzyme does not. The optimal conditions for the PSC1 enzyme, including pH and temperature were determined. The L-phenylalanine DOT-T1E derivative *Pseudomonas putida* CM12-5 that overproduces L-phenylalanine was used as the host for expression of *pal/psc1* genes to efficiently convert L-phenylalanine into *t*CA, and the aromatic carboxylic acid into styrene. The highest styrene production was achieved when the *pal* and *psc1* genes were co-expressed as an operon in *P. putida* CM12-5. This construction yielded styrene production exceeding 220 mg L^−1^. This study serves as a successful demonstration of our strategy to tailor functional enzymes for novel host organisms, thereby broadening their metabolic capabilities. This breakthrough opens the doors to the synthesis of aromatic hydrocarbons using *Pseudomonas putida* as a versatile biofactory.

**Supplementary Information:**

The online version contains supplementary material available at 10.1186/s12934-024-02341-0.

## Highlights

1. This study focuses on the conversion of sugars into styrene, a valuable chemical compound, using as a host the solvent-tolerant *Pseudomonas putida* DOT-T1E as a chassis.
2. The biosynthesis of styrene involves a two-step process, starting with the conversion of L-phenylalanine into *trans*-cinnamic acid (*t*CA) through PAL enzymes, followed by decarboxylation to yield styrene.
3. A synthetic *trans*-cinnamic acid decarboxylase was designed using a novel 'wholesale' approach that involved the derivation of consensus sequences from homologous yeast FDC1 genes with > 60% identity, leading to the design of a functional *Pseudomonas* codon-optimized protein named PSC1.
4. Assays demonstrated successful decarboxylation of *t*CA into styrene by the PSC1 enzyme.
5. Optimal conditions for PSC1 enzyme activity in vivo were determined, including pH and temperature. Highest styrene biosynthesis efficiency was achieved by co-expressing the *pal* and *psc1* genes as an operon in *P. putida* CM12-5, a *P. putida* DOT-T1E derivative that produces L-phenylalanine, showcasing the significance of coordinated expression for improved chemical production.
6. The utilization of the solvent-tolerant *Pseudomonas putida* chassis as a biofactory for styrene production highlights the potential of microbial engineering for sustainable and environmentally-friendly chemical synthesis.

**Supplementary Information:**

The online version contains supplementary material available at 10.1186/s12934-024-02341-0.

## Introduction

Styrene is one of the most widely used starting materials for the production of plastics. It is used to make solid polystyrene, polystyrene copolymers, rubber, composites and many others [1–3]. Current demand for styrene is estimated to be about 30 million metric tons per year [4]. It is mainly produced through chemo catalysis by the dehydrogenation of petroleum-derived ethylbenzene [5]. This process requires about 3 metric tons of steam per metric ton of styrene, making styrene the most energy intensive of all commodities derived from petrol [6]. Because of this, new more environmentally friendly approaches are needed for synthesis of this aromatic hydrocarbon.

**Fig. 1:**
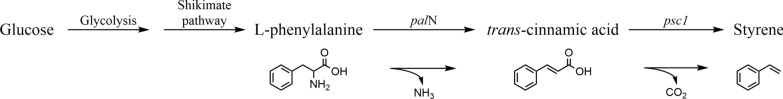
Enzymatic pathway to produce styrene from glucose, via the intermediates L-phenylalanine and *trans*-cinnamic acid. The two-step pathway from L-phenylalanine to styrene is achieved by co-expressing *pal*N, encoding a phenylalanine ammonia lyase, and *psc1*, encoding a *trans*-cinnamic acid decarboxylase

An alternative approach to chemical synthesis relies on biorefineries that can produce styrene at room temperature and ambient pressure from sugars. Styrene can be biosynthesized from L-phenylalanine (L-Phe) through two enzymatic steps (Fig. 1). First, a reaction catalyzed by phenylalanine ammonia lyase (PAL) converts L-Phe into *trans*-cinnamic acid (*t*CA) through its non-oxidative deamination [7–12]. Next, *t*CA is decarboxylated to styrene—a reaction that has been described in fungi and which is catalyzed by ferulic acid decarboxylases [13].

McKenna and Nielsen [3] tested a wide range of PAL enzymes from eukaryotic and prokaryotic origin and showed that PAL enzymes from the cyanobacteria *Nostoc punctiforme* and *Anabaena variabilis* [8, 11] were highly specific for L-Phe deamination, and stoichiometrically converted L-Phe into *t*CA. McKenna and Nielsen [3] also showed that the yeast ferulic acid decarboxylase FDC1 can convert *t*CA into styrene. Furthermore, it is known that a PAL/FDC1 pathway operates in *Escherichia coli* and yeast*;* allowing production in the range of 29 to 260 mg L^−1^ [3, 14]. The limitations in styrene production seem to arise from the inherent toxicity of styrene, which results from its tendency to partition to cell membranes, where it disrupts membrane structure and proton gradients, leading to cell energy collapse and cell death [15–17].

*Pseudomonas putida* DOT-T1E is extremely tolerant to styrene as the strain is able to grow in a second phase of this aromatic hydrocarbon as well as other toxic compounds such as toluene [16, 18]. Tolerance of DOT-T1E to these chemicals is the result of a number of adaptations, which include: (1) the presence of a series of efflux pumps that remove solvents from cell membranes and the periplasmic space [19, 20]; (2) the ability to strengthen membranes via phospholipid adjustments, such as the *cis* to *trans* isomerization of fatty acid chains and the biosynthesis of cardiolipin as a head group [16, 21, 22]; (3) the presence of a series of chaperones that help to fold newly synthesized proteins [18]; (4) the expression of a number of oxidative stress proteins that can quench the reactive oxygen species that result from the uncoupling of respiratory chains [16, 18, 20, 21, 23]; and (5) an enhanced ability to generate energy through robust carbon metabolism fluxes. In addition, previous assays had shown that DOT-T1E does not use *t*CA or styrene as a C-source [16, 24].

Given these characteristics, along with the fact that the metabolic map of the strain is known [24] and the ease with which *P. putida* DOT-T1E can be genetically manipulated, this strain has gained interest as a platform for the biosynthesis of highly toxic aromatic compounds. Godoy et al. [25] and Molina Santiago et al. [26] described strain CM12-5, a *P. putida* DOT-T1E derivative which can be used as a chassis for production of aromatic compounds from L-Phe. This mutant produces excess of L-Phe and was generated through chemical mutagenesis and inactivation of a series of L-Phe metabolism genes. The genes that were mutated in CM12-5 were: T1E_0122 and T1E_3356 (4-hydroxyphenylpyruvate dioxygenases involved in transformation of phenylpyruvate to 2-hydroxyphenylpyruvate), T1E_4057 (a phenylalanine 4-monooxygenase involved in transforming phenylalanine to tyrosine), T1E_1753 (an enzyme that converts phenylalanine to 2-phenylacetamide) and T1E_1616 (an aldehyde dehydrogenase that transforms phenylacetaldehyde to phenylacetate). This *P. putida* CM12-5 mutant was found to be able to produce excess of L-Phe that was excreted to the medium [26].

Based on findings from McKenna and Nielsen [3] that cyanobacterial PAL enzymes can stoichiometrically transform L-Phe into *t*CA, these genes were incorporated in the *P. putida* CM12-5 chassis. When a PAL enzyme is incorporated in the genetic background of the *P. putida* CM12-5 strain, it effectively converted L-Phe into *t*CA [26]. The modified strain was able to synthesize *t*CA at 190 mg per liter in 48 h of growth [26]. While these findings are promising, the key remaining challenge for the efficient biosynthesis of styrene is the need for a functional *trans*-cinnamic acid decarboxylase in this chassis. The only known enzyme able to convert *trans*-cinamic acid into styrene are fungal ferulate decarboxylases, but expressing eukaryotic genes in prokaryotes in general, and *Pseudomonas* in particular, presents consistent challenges, due to (i) the inherent disorganization of eukaryotic proteins compared to their prokaryotic counterparts; (ii) eukaryotic proteins often require chemical modifications for activation or to achieve optimal activity, processes not typically carried out by prokaryotes; and (iii) the inappropriate expression of eukaryotic genes may lead to the formation of aggregates, such as inclusion bodies of the recombinant protein [27]. Recent progress in overcoming these challenges has been achieved through the development of optimized genes. This includes leveraging host codon preferences, designing vectors with modulable promoters, and refining culture conditions [28]. While significant strides have been made in this field, specific obstacles still persist.

A notable recent advancement in protein design involves the expression of “consensus” sequences, as detailed by Sternke et al. [29, 30]. These researchers proposed that consensus-derived proteins, utilizing a “wholesale” approach, encapsulate the evolutive trajectory of a group of proteins with conserved domains or regions. The consensus design involves creating a sequence based on the most frequent residues in a multiple sequence alignment of proteins from the same family. Despite differences in overall residue composition from naturally occurring sequences, consensus proteins have been described to be active and thermodynamically stable. The conservation of residues in active sites and binding interactions interfaces in consensus sequences, are key in designing active proteins derived from consensus [30]. In fact, these authors explored across several protein families and found that their consensus folded well and some displayed increased thermodynamic stability compared to natural homologs.

Here, we address the challenge of designing in silico and synthesizing in vitro a gene that gives rise to a functional *trans*-cinnamic acid decarboxylase (PSC1) that converts the aromatic carboxylic acid in styrene when expressed in *P. putida*. Furthermore, when the *psc*1 gene was co-expressed with a *pal* gene in the solvent-tolerant L-phenylalanine overproducer *P. putida* CM12-5 strain, styrene from glucose was produced in minimal medium, paving the way for the sustainable production of this valuable aromatic hydrocarbon.

## Materials and methods

### Chemicals

Chemicals used in this study were purchased from Sigma-Aldrich and include L-Phe, *t*CA, styrene, acetonitrile and phosphoric acid.

### Bacterial strains, plasmids and growth conditions

Strains and plasmids used in this study are listed in Table 1. *Pseudomonas putida* strains were grown on M9 minimal medium with glucose 5 g L^−1^ [31] as the sole carbon source. When required, different concentrations of L-Phe or *t*CA were supplied. Cultures were incubated at 30 °C and shaken on an orbital platform at 200 strokes per minute (unless otherwise indicated). *Escherichia coli* DH5α was used for cloning experiments and cells were grown on LB at 37 ºC. Growth of liquid cultures was determined by following the turbidity of (OD_660_) of the cultures. Antibiotics were added, when needed, to reach the following final concentrations: 100 μg mL^−1^ ampicillin (Ap), 25 μg mL^−1^ kanamycin (Km), 10 μg mL^−1^ gentamycin (Gm) and 10 μg mL^−1^ rifampicin (Rif). When indicated, 1 mM 2-methylbenzoate (2-MB) was added to the medium.

**Table 1:** Bacterial strains and plasmids used in this study

Strain or plasmid	Characteristics	References
Strains		
*Pseudomonas putida*		
DOT-T1E	Rif^R^, Tol^R^	[27]
CM12-5	Rif^R^, Tol^R^, overproduces L-Phe	[25, 26]
*Escherichia coli*		
DH5α	Cloning host for pSEVA plasmids	[28]
Plasmids		
pSEVA238	Expression vector; *ori*pBBR1, *xylS*-Pm, Km^R^	[29]
pPALN	pSEVA238 derivative carrying *pal* genes from *Nostoc punctiforme* and *Streptomyces maritimus*	[26]
pPALN_C1	pSEVA238 derivative carrying *pal* genes from *Nostoc punctiforme* and *Streptomyces maritimus* and *psc1* gene	This work
pSEVA632	Expression vector; *ori*pBBR1, Gm^R^	[29]
pPSC1	pSEVA632 derivative carrying *psc1* gene	This work
pPSD1	pSEVA632 derivative carrying *psd1* gene	This work

Rif^R^, rifampicin-resistant; Gm^R^, gentamycin-resistant; Km^R^, kanamycin-resistant; Tol^R^, toluene tolerant; L-Phe, L-phenylalanine; *ori*pBBR1, origin of replication pBBR1; *xylS*-Pm, XylS-Pm regulator/promoter system

### DNA techniques

DNA was manipulated using standard laboratory protocols [32, 33]. Genomic DNA was isolated using the Wizard Genomic DNA purification Kit (Promega USA), while plasmid DNA was isolated with the QIAprep Spin Miniprep kit (Qiagen, USA). DNA concentration was measured with a NanoDrop One (Thermo Scientific, USA). PCR DNA amplification was performed with universal primers, dNTPs and Phusion High-Fidelity DNA polymerase (Thermo Scientific, USA) or Taq DNA polymerase (Roche, Germany), as recommended by the manufacturers.

### Electroporation

Electroporation of *Pseudomonas putida* strains was performed as described elsewhere [32, 33], using a MicroPulser electroporator and Gene Pulser Cuvettes with 0.2 cm gap (Bio-Rad, USA). Transformants were selected on LB agar plates with kanamycin or gentamycin and incubated at 30 °C for 24 h.

### In silico identification of sequences with high similarity to the *S. cerevisiae* enzyme *trans*-cinnamic acid decarboxylase 1 (FDC1)*.* Design and in vitro synthesis of FDC1-like enzymes

The *Saccharomyces cerevisiae* FDC1 sequence was used as a query to identify in UniProtKB homologous sequences using BLAST [34]. UniProt KB was used because it is a large resource of protein sequences with detailed annotations and cross-references to external data collection such as DDBJ/EMBL/GenBank [35]. Multiple alignments of different homologous sequences were performed using the MultAlin program (http://multalin.toulouse.inra.fr/multalin/). A consensus sequence from each multiple alignment was derived.

Once the protein consensus sequences were obtained, the synthesis of the corresponding *Pseudomonas* codon-optimized genes was carried out by GenScript^®^. The synthetic genes (*psc1* and *psd1*) were cloned into the Gm^R^, broad-host range pSEVA632 plasmid [36] flanked by enzyme restriction sites (BamHI and EcoRI) and transformed into *E. coli* DH5α cells. Next, the resulting plasmids pPSC1 and pPSD1 were transformed into *P. putida* DOT-T1E, *P. putida* CM12-5, and *P. putida* CM12-5 (pPALN) strains. The correct cloning of the synthetic variants was confirmed by DNA sequencing. To construct pPALN_C1, the *psc1* gene was sub-cloned from pPSC1 (BamHI and EcoRI fragment) into pPALN downstream the *pal* genes.

### PAL activity assay in *P. putida* whole cells

The activity of PAL was tested at different pH and temperatures. To this end, *P. putida* CM12-5 (pPALN) cells were grown in M9 minimal medium with glucose as the carbon source. At OD_660_ 0.4 to 0.6, 1 mM 2-MB was added to the cultures to induce expression of the *pal* genes from the XylS regulated Pm promoter [37]. At OD_660_ 1, the cultures were concentrated to an OD_660_ of 10 in 4 mL of M9 medium without glucose, supplemented with 100 mg L^−1^ of L-Phe. The cultures were incubated in test tubes for 24 h at 200 rpm, within a temperature range of 18 to 37 ºC and a pH range of 5.8 to 7.6. Supernatant aliquots were taken at different time points between 0 and 24 h for metabolite analysis. Samples were prepared by removing 1 mL of culture from test tubes and pelleting the cells at 11,000 ×*g* for 4 min. The supernatant (0.75 mL) was then transferred to a glass vial for HPLC analysis of L-Phe utilization and *t*CA production. Initial consumption rates (mg L^−1^ h^−1^) were determined by calculating the slope of a trendline in the first two hours of the assay.

### *Trans*-cinnamic acid decarboxylase activity assay in *P. putida* whole cells

The procedure was carried out as described above except that the strain used bore the pPSC1 or pPSD1 plasmid and the substrate used was 100 mg L^−1^ of *t*CA. Previously, we tested the functionality of the synthetic genes (*psc1* and *psd1*). To this end, *Pseudomonas putida* CM12-5 transformants bearing a plasmid encoding the PSC1 or PSD1 protein were grown in M9 minimal medium with glucose as the sole C-source in the presence of 0.25, 0.5 or 1 mM *t*CA.

### Styrene production from glucose by *Pseudomonas putida*

The strains *P. putida* CM12-5 (pPALN, pPSC1) and *P. putida* CM12-5 (pPALN_C1) were tested for styrene production. The procedure was as follows: cells were grown in M9 minimal medium with glucose as the carbon source. At OD_660_ 0.4–0.6, 1 mM 2-MB was added to the cultures and at OD_660_ 1, the cultures were concentrated to an OD_660_ of 10 in 4 mL of the same medium (pH 7). The cultures were incubated for 62 h in 20 mL gas-tight HS vials, sealed to prevent styrene losses, at 30 ºC and 200 rpm. Then, total styrene content was analyzed as described below. Simultaneously, additional replicates were set up to collect supernatant aliquots and determine the concentrations of glucose, L-Phe, and *t*CA.

#### Metabolite analysis

L-Phe and *t*CA levels were determined in culture supernatants using an Agilent/HP 1050 HPLC System (Agilent/HP, USA), equipped with a Nova-Pak C18 column (4 μm, 3.9 mm × 150 mm, Waters) and coupled to a DAD detector. Milli-Q H_2_O acidulated with 0.1% (v/v) H_3_PO_4_ (A), and acetonitrile: H_2_O (90:10, v/v) supplemented with 0.1% H_3_PO_4_ (B) were used as eluents. Samples (20 μL) were injected for analysis at a constant flow rate of 1 mL min^−1^ for isocratic separation using a mixture of 40% (v/v) A and 60% (v/v) B. When an elution gradient was required, the same eluents were used with the following ramp of solvents and times: the method started with 2 min 95% A; then, mobile phase changed to 20% B within 8 min. Finally, the mobile phase was returned to the initial conditions in a 3 min hold time. Column temperature was 20°C. Under these conditions, the eluent was monitored at 215 nm for L-phenylalanine and at 280 nm for *t*CA.

Glucose determination in supernatants was carried out using the D-glucose-HK Assay Kit (Megazyme, Ireland) according to manufacturer’s instructions. Absorbance measurements were carried out using a TECAN Sunrise 200 microplate absorbance reader (Tecan GmbH, Austria).

#### Styrene analysis

Styrene measurements were performed by HS-SPME coupled to GC–MS. The chromatographic separation was carried out using an Agilent 7890A gas chromatograph (Agilent, USA) with a Zebron™ ZB-5MS column (30 m, 0.23 mm ID, 0.23 μm film; Phenomenex, USA). Helium gas was used as carrier gas at a 1.2 mL min^−1^ flow rate. The samples were injected in split mode (100:1) and the injector temperature was held at 240 ºC. The column temperature program started at 40 ºC for 2 min and then ramped up to 240 ºC at 10 ºC per min and held there for 2 min. A mass spectrometer detector was used (model Quattro micro GC; Waters, USA) with an electron impact ionization source of 70 eV. The temperatures of the MS source and MS transfer line were set at 240 ºC.

SPME fiber assembly Divinylbenzene/Carboxen/Polydimethylsiloxane (DVB/CAR/PDMS, 50/30 μm, 23 ga) was used for the HS-SPME procedure. The pre-incubation time was 3 min. The incubation was carried out at 40ºC and 500 rpm for 15 min. The desorption temperature was 240 ºC and the desorption time was 2 min.

Styrene and other biotransformation products present in the samples were unequivocally identified by matching the corresponding mass spectra with a standard NIST17 spectral database.

### Phylogenetic tree

We constructed a phylogenetic tree to understand the relationships between the sequences with > 50% identity with FDC1 from *Saccharomyces cerevisiae* (see Additional file 1: Fig. S1)*.* Sequences were aligned with Muscle software and served as input to construct the phylogenetic tree with iqtree software v 1.6.12 (parameters -nt AUTO, -bb 1000 -m TESTMERGE -safe) [38, 39]. The maximum likehood tree was constructed following the LG + I + G4 evolution model, which was the best fit according to ModelFinder [40], with 1000 bootstrap replicates. Finally, the phylogenetic tree was plotted using iTOL v6 software.

### Statistical analysis

Two-tailed Student’s t-tests were performed to determine the statistical significance for two-group comparisons. For three-group comparisons, ANOVA analysis was conducted. If statistical significance was found, the Tukey test was performed between paired groups. Differences were considered to be significant if a *p*-value < 0.05 was obtained.

## Results

### Design and synthesis of a functional *trans*-cinnamic acid decarboxylase in *P. putida*

The expression of eukaryotic genes in *Pseudomonas putida* is a challenge, and when expression is achieved, the resulting proteins may not be functional or exhibit low activity as it was the case when a *Pseudomonas* codon-optimized yeast FDC enzyme was expressed in *P. putida* (not shown). We then searched in NCBI and other databases for prokaryotic enzymes similar to *Saccharomyces cerevisiae* FDC1; however, no results were returned. We also carried out BLAST searches using the FDC1 enzyme sequence in the UniProtKB database [35]. This returned a limited number of eukaryotic proteins with identities ranging from 88.9 to 50% (see Additional file 5: Table S1) and their phylogenetic relationship is shown in Additional file 1: Fig. S1. To avoid synthesizing and testing a number of codon-optimized eukaryotic genes in *P. putida*, we carried out de novo design of a protein using the ‘wholesale’ method. This involves deriving a consensus sequence from the multi-alignment of related proteins. Sternke et al. [29] revealed that this strategy results in proteins with high stability because the consensus sequence consolidates the evolutionary history of the protein to yield enhanced stability and functionality. We used two series of homologous FDC1 sequences, which were stratified according to percentage of identity. The alignment of the proteins with > 60% identity (8 sequences) and > 50% identity (74 sequences) to FDC1 from *Saccharomyces cerevisiae* is shown in Additional file 2: Fig. S2 and Additional file 3: Fig. S3, respectively. A consensus sequence for each of the two series was derived. At each position in these sequences, the chosen amino acid was the most probable one based on frequency of appearance. When the program did not derive a residue, the language system shown in Additional file 6: Table S2 was used.

When designing polypeptide sequences, the amino acid that was present in the highest proportion was chosen. When proportions were the same for two amino acids, either of the two possible amino acids was selected indiscriminately. When no amino acid was present in higher proportion, we attempted to maintain the protein's charge as neutral as possible by choosing either a polar, uncharged amino acid (i.e., asparagine (N) or glutamine (Q)). For gaps in aligned sequences—when the alignment resulted in the absence of an amino acid—that residue was eliminated. However, if the gap was due to non-matching amino acids, the one present in the highest proportion was selected. If there was no consensus amino acid, to fill in gaps rather than predicting the most probable residue based on language models [41] we opted to incorporate alanine as it is a neutral amino acid whose small volume is known to minimally affect protein folding [42, 43]. The amino acid consensus sequences (Additional file 2: Fig. S2 and Additional file 3: Fig. S3) were converted into *Pseudomonas* codon-optimized genes that we named *psc*1 (for proteins with > 60% identity; Additional file 7: Table S3) and *psd*1 (for proteins exhibiting > 50% identity; Additional file 8: Table S4). The genes were synthesized by GenScript^®^, cloned into the broad-host range pSEVA632 (Gm^R^) plasmid and transformed into *E. coli* DH5α and *P. putida* CM12-5.

### In vivo assay of a synthetic *trans*-cinnamic acid decarboxylase in *P. putida*

**Fig. 2:**
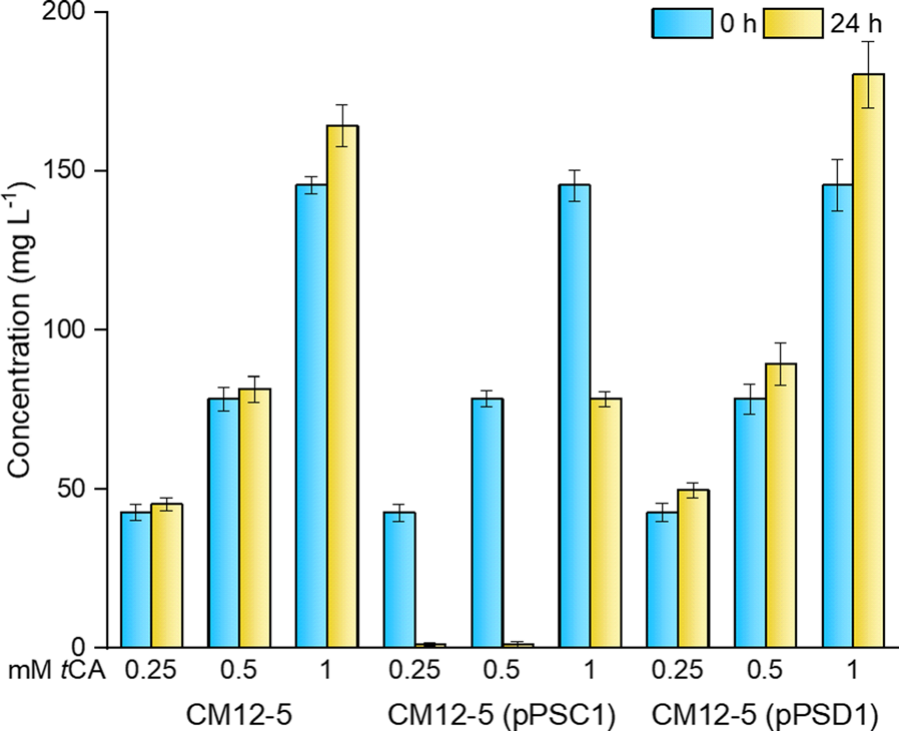
*Trans*-cinnamic acid consumption by *P. putida* CM12-5 expressing different *trans*-cinnamic acid decarboxylases. *P. putida* CM12-5 without plasmid or bearing the plasmid pPSC1 or pPSD1 were grown on M9 minimal medium with glucose supplemented with 0.25, 0.5 or 1 mM *trans*-cinnamic acid. *t*CA concentrations were determined at the beginning of the assay (blue bars) and after 24-h cultivation (yellow bars). ANOVA analysis was performed for three-group comparisons and Tukey test was carried out between paired groups to determine the statistically significance (*p*-value < 0.05). The results shown are the averages and standard deviations of three independent assays

*Pseudomonas putida* CM12-5 transformants bearing a plasmid encoding the PSC1 or PSD1 protein were grown in M9 minimal medium with glucose as the sole C-source in the presence of 0.25, 0.5 or 1 mM *t*CA. We found that *t*CA levels did not decrease when *Pseudomonas putida* CM12-5 strain expressed the PSD1 enzyme as was the case of the control without a *fdc* gene; however, when strain carried the PSC1 enzyme, *t*CA disappeared from the medium after a 24 h incubation with 0.25 or 0.5 mM *t*CA. When 1 mM *t*CA was added, its concentration dropped by half within the same timeframe (Fig. 2). At all *t*CA concentrations tested, the differences in *t*CA concentration at 24 h were statistically significant between PSC1 and PSD1 (p < 0.05).

This revealed that PSC1 is a novel functional enzyme capable of metabolizing *t*CA. As expected, at any concentration of *t*CA and with both constructions, L-Phe accumulated in culture medium as its metabolism is blocked in the CM12-5 mutant strain (data not shown), which supports the hypothesis that the metabolism of *t*CA does not interfere with the general physiology of the strain.

**Fig. 3:**
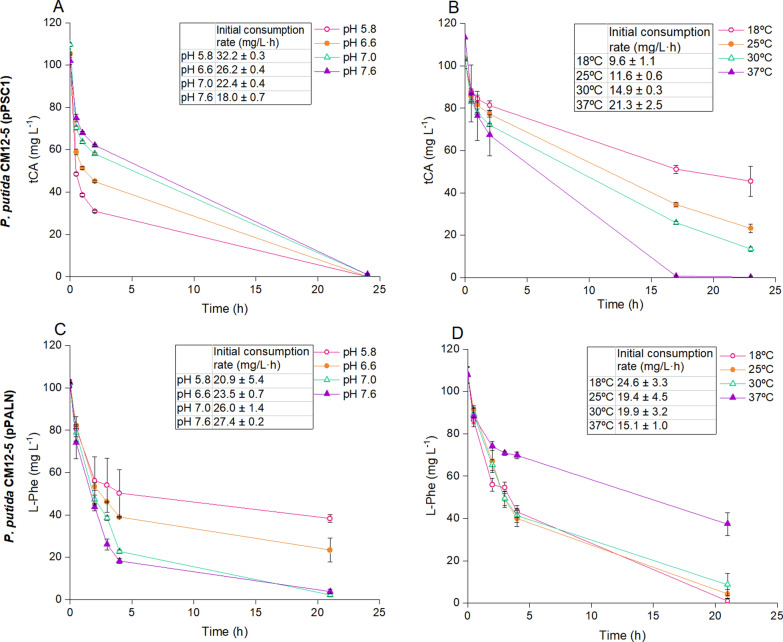
*Trans*-cinnamic acid and L-phenylananine consumption by *P. putida* CM12-5 (pPSC1) (**A** and **B**) and *P. putida* CM12-5 (pPALN) (**C** and **D**), respectively, at different pH and temperatures. For pH assays (**A** and **C**), cultures were grown at pH 5.8, pink open circles; pH 6.6, orange solid circles; pH 7.0, green open triangles; and pH 7.6, purple solid triangles. For temperature assays (**B** and **D**), cultures were grown in M9 minimal medium at pH 7.0 at 18 °C, pink open circles; 25 °C, orange solid circles; 30 °C, green open triangles; and 37 °C, purple solid triangles. Initial consumption rates correspond to the slope of a trendline during the first two hours of cultivation. The results shown are the averages and standard deviations of three independent assays

Next, we tested the conversion of *t*CA into styrene under different growth conditions. As expected, the control—*P. putida* CM12-5 (pPSC1) in the absence of *t*CA—did not produce styrene in medium with glucose; however, when 0.25 mM *t*CA was added, a single conversion peak was recorded at 24 and 48 h. The mass spectra of this peak was compared to the NIST17 spectral Database, which unequivocally identified it as styrene (Additional file 4: Fig. S4), confirming that styrene is biosynthesized by the strain from *t*CA. We determined the conversion rate of *t*CA under different temperatures and pH, using resting cell assays with cells suspended at a OD_660_ of 10 in M9 minimal medium without glucose but containing 100 mg L^−1^ *t*CA. The chosen pH range covered values between 5.8 and 7.6, which align with the pH range for the growth of *P. putida* [44]. We found significant higher conversion rates at pH 5.8 and 6.6 (26.2–32.2 mg L^−1^ h ^−1^) than at pH values that exceeded 6.6 (Fig. 3A). *P. putida* thrives at temperatures from 18 to 37ºC, and the optimal temperature for conversion was 37ºC with lower rates of transformation at 18ºC (Fig. 3B).

### Conversion of L-phenylalanine into *trans*-cinnamic acid

Because production of styrene will require coupling of the whole set of reactions from glucose to L-Phe, we transformed CM12-5 with the construct made by Molina-Santiago et al. [26], which expresses the *pal* genes from the Pm promoter. We cultured *P. putida* CM12-5 (pPALN) on glucose as the sole C-source in the absence and in the presence of 1 mM L-Phe. As expected, we found that *P. putida* CM12-5 without the *pal* genes accumulated L-Phe in the culture medium over time, reaching 217.2 ± 41.8 mg L^−1^ in the absence of the amino acid. Spiking L-Phe to a level of 170 mg L^−1^ at t = 0 did not prevent accumulation of this amino acid, reaching a final concentration of 356.9 ± 5.5 mg L^−1^ of L-Phe (Table 2). This indicated that intracellular accumulation of L-Phe from glucose was not subject to feedback inhibition by this aromatic amino acid. When *pal* genes were expressed in the *P. putida* CM12-5 strain, L-Phe transiently accumulated and *t*CA was detected in the culture medium, reaching a concentration of 91.0 ± 19.2 to 199.7 ± 19.3 mg L^−1^ (Table 2). As above, the presence of excess L-Phe did not result in inhibition of the synthesis of *t*CA or L-Phe.

**Table 2:** Biosynthesis of L-phenylalanine and *trans*-cinnamic acid by *P. putida* CM12-5 and *P. putida* CM12-5 (pPALN)

	[L-Phe] _T 0 h_ (mg L^−1^)	[L-Phe] _T 24 h_ (mg L^−1^)	[*t*CA] _T 24 h_ (mg L^−1^)
CM12-5	0	217.2 ± 41.8	0
	173.2 ± 0.9	356.9 ± 5.5	0
CM12-5 (pPALN)	0	141.8 ± 33.8	91.0 ± 11.2
	173.6 ± 0.1	263.9 ± 41.0	119.7 ± 19.3

Assays were done in M9 minimal medium with glucose as the sole C-source with and without the addition of 1 mM L-Phe (173 ± 1 mg/L)

The concentrationof L-phenylalanine and *trans*-cinnamic acid was determined after 24 h’ cultivation

The data in the table are the average and standard deviation of three independent assays

PAL activity was analyzed using resting cell assays with cells suspended at a OD_660_ of 10 in M9 minimal medium without glucose but containing 100 mg L^−1^ L-Phe. The rates of L-Phe consumption during the first two hours of cultivation at pH 6.6 and 7.6 were 23.5 ± 0.7 to 27.4 ± 0.2 mg L^−1^ h^−1^ and the production of *t*CA was 22.5 ± 0.6 to 26.6 ± 0.7 mg L^−1^ h^−1^ with > 97% of L-Phe converted into *t*CA. At pH of 5.8 we also found stoichiometric conversion of L-Phe into *t*CA*,* but the rate of accumulation was half of that at neutral pH (Fig. 3C). At pH 7 the effect of temperature between 18 and 37ºC was tested (Fig. 3D). Maximal rates were observed in the range between 18 and 30 ºC, with lower activity at 37 ºC and eventual cessation of *t*CA production in agreement with the limited viability of *P. putida* at the highest temperature tested.

At pH 7.0 and 30ºC, all L-Phe was converted into *t*CA. These results are in line with earlier studies, which show that the first step in the pathway is highly specific for the intended L-Phe substrate—a fact that will be advantageous for controlling the synthesis of aromatic hydrocarbons (i.e., styrene) from sugars.

### Styrene biosynthesis from glucose

**Fig. 4:**
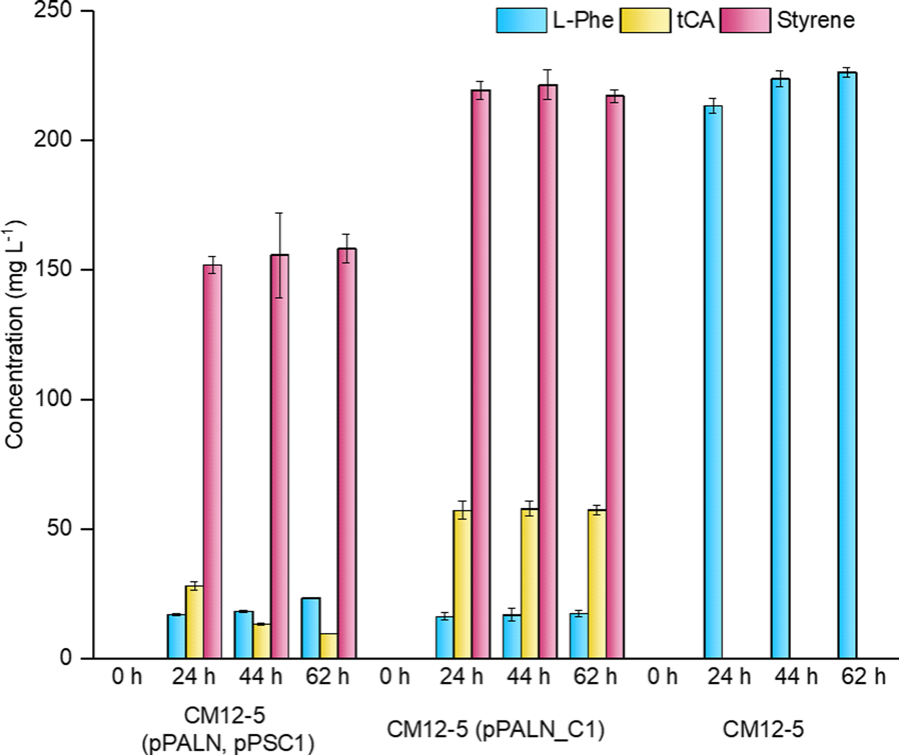
Biosynthesis of styrene by *P. putida* derivatives from glucose. The strains used were *P. putida* CM12-5 (pPALN, pPSC1)*, P. putida* CM12-5 (pPALN_C1) and *P. putida* CM12-5 as control. Cells were grown on M9 minimal medium with 0,5% (w/v) glucose as the sole C-source. Production of L-phenylalanine (blue bars), *trans*-cinnamic acid (yellow bars) and styrene (pink bars) was determined. Two-tailed Student’s t-tests were performed to determine the statistical significance for two-group comparisons (*p* < 0.05). The results shown are the averages and standard deviations of three independent assays

Next, we investigated the bioconversion of glucose in styrene using *P. putida* CM12-5 (pPALN, pPSC1). These assays were conducted in M9 minimal medium with 0.5% (w/v) glucose at pH 7.0 and 30 °C. We selected these conditions because at this pH and temperature, the in vivo performance of both enzymes, although not reaching their highest activity levels, approached their optimal activity range (see Fig. 3). After culturing for 24 h, we measured the production of 158 ± 6 mg L^−1^ of styrene. It should be noted that only a small proportion of L-Phe and *t*CA remained in the supernatants (Fig. 4). As expected, the control strain *P. putida* CM12-5, did not produce styrene, and only accumulated L-Phe in supernatants.

In an effort to enhance styrene production, the broad-host range plasmid pPALN_C1 (Table 1) bearing both *pal* genes and *psc1* gene in tandem was constructed. The genes were expressed under the XylS-dependent Pm promoter in the presence of 1mM 2-methylbenzoate to maximize expression levels as decribed before by Ramos et al. [37]. The recombinant *P. putida* CM12-5 (pPALN_C1) was grown in M9 minimal medium with 0.5% (v/v) glucose as described above. Under these conditions the strain produced 221 ± 6 mg L^−1^ styrene (Fig. 4), showing significant superior efficiency (*p* < 0.05) compared to the *P. putida* CM12-5 (pPALN, pPSC1) strain, the estimated yield was 44 ± 1 mg styrene per g glucose.

## Discussion

*Pseudomonas putida* DOT-T1E and its derivative CM12-5 are highly solvent tolerant strains, that can thrive in the presence of aromatic hydrocarbons such as toluene, xylene, ethylbenzene and styrene, and they are considered as useful chassis for the biosynthesis of aromatic compounds [45]. These strains acquired tolerance to these compounds through adjustments made in the phospholipid composition of cell membranes, increased robustness of the protein folding machinery, and a set of efflux pumps that extrude solvents to the external environment from the cytoplasm, periplasm and cell membranes [23]. In addition to solvent tolerance, a crucial characteristic of microbial chassis for chemical production [46, 47] is the ability to maintain the integrity of the desired product(s). In the case of *P. putida* CM12-5 strain, a phenylalanine producing strain catabolism of the aromatic amino acid is blocked, in addition, the strain does not metabolize *t*CA and styrene, compounds that can be made from L-phenylalanine [16]. This makes this strain a promising candidate for the synthesis of these aromatic compounds.

In the current solvent-tolerant platform we used a two step conversion of L-phenylalanine into styrene (Fig. 1). In the initial step, we utilized PAL enzymes, which have been demonstrated by McKenna and Nielsen [3] to efficiently catalyze this reaction (Fig. 1). The second step in the proposed styrene biosynthesis pathway involves the decarboxylation of *t*CA by a ferulic decarboxylase (FDC), which exhibits *trans*-cinnamate decarboxylase activity and results in the production of styrene (Fig. 1). Our approach to design a functional *trans*-cinnamic decarboxylase was based on Sternke et al. [29] that proposed that consensus sequences derived from multi-alignment of protein families, in the so called “wholesale” approach, yield functional and thermodynamically stable proteins. Following several multi-alignments of FDC proteins, consensus sequences were derived and the protein translated to DNA with *Pseudomonas* codon optimized sequence. From the alignment of 8 FDC sequences with > 60% identity to the *S. cerevisiae* enzyme we derived PSC1 that was shown to be functional in *P. putida*. The PSC1 enzyme consists of 502 amino acids and differs by 114 residues with respect to the FDC1 from *S. cerevisiae*. Another variant, named PSD1, differs by 230 residues from the PSC1 enzyme and 223 residues from the FDC1 of *S. cerevisiae*, and it was found to be non functional. Studies by Bailey et al. [48] and Duță et al. [49] demonstrated that specific residues (I189, Q192, I330, F397, I398) in the *S. cerevisiae* FDC1 enzyme are crucial for substrate binding/catalysis, and these residues are conserved in the PSC1 enzyme. However, in the PSD1 protein sequence, F397 was replaced by Y and I398 by T. Whether these residues are responsible for the lack of activity of the PSD1 enzyme remains unknown. Our current results suggest that the PSC1 is a dimeric enzyme that utilizes the atypical prenylated flavin mononucleotide as a co-factor (García-Franco et al. unpublished).

In the pursuit of establishing a platform for the bioproduction of styrene as a sustainable alternative to petroleum-derived styrene, a high production titer is needed. Previous studies by Lee et al. [50] and Grubbe et al. [51] underscored this necessity. In our assays *P. putida* CM12-5 derivatives carrying the *palN/psc1* operon exhibited production titers of 221 ± 6 mg L^−1^, and our results support that almost 100% of L-Phe produced by CM12-5 (pPALN_C1) was eventually converted into styrene, with a minor fraction transiently accumulating as *t*CA when the process initiated from glucose. This also indicates that the majority of the synthesized *t*CA is rapidly converted to styrene. Furthemore, additional assays supplementing *P. putida* CM15-2 (pPALN_C1) suspensions with exogeneous L-Phe (i. e. 1 mM) confirmed its complete conversion to styrene. Consequently, our findings pinpoint that the limiting step in the production of styrene is the intracellular production of L-phenylalanine, which emphasizes the need to increase L-Phe yields in this platform.

Our assays revealed that co-expressing the *pal* and *psc1* genes as an operon led to a 25% increase in styrene production, compared to the levels achieved when the genes were expressed individually in two different plasmids. This enhanced production probably relates to (i) reduced basal maintenance energy for cells with a plasmid versus strains with two plasmids that need two antibiotics for selection; and (ii) a more efficient conversion of L-Phe into *t*CA and styrene, thereby preventing the accumulation of the amino acid, it is known that excess L-Phe inhibits of prephenate dehydrogenase (PheA), an enzyme crucial in L-Phe biosynthesis [26, 52]. Therefore, the controlled expression of genes involved in the biotransformation process of styrene biosynthesisis is critical.

Molina-Santiago et al. [26] described that a *P. putida* CM12-5 derivative produced a maximum titer of approximately 600 mg L^−1^ L-Phe after 24 to 48 h of culture in M9 with 1.5% (w/v) glucose. Since stoichiometric conversion of L-phenylalanine into styrene takes place, we assumed that in conditions in which 600 mg L^−1^ of L-Phe are reached, its complete conversion to styrene would lead to a production of 378 mg L^−1^ of styrene, surpassing the solubility of styrene in water. Our earlier study[16] indicated that the growth rate and viability of *P. putida* DOT-T1E are only marginally lower in the presence of styrene compared to growth without solvent [16]. Hence, we hypothesize that the balance of host fitness would probably remain unaffected by the potentially achievable titer of 378 mg L^−1^. Nonetheless, implementing strategies to recover styrene concurrently during production could prove valuable in mitigating any metabolic burden of solvent tolerance, thus allowing for resource redirection to maximize styrene yields. Since our system attains maximum titer after 24h cultivation, adopting for instance a fed-batch system with advanced recovery and gas stripping techniques could potentially yield styrene productivity comparable to that achieved in the studies carried out by Nielsen’s group, i.e. 836 mg L^−1^ [3, 50, 51, 53–55]. Similarly to our earlier research [25], approximately 12% of available sugars in lignocellulose wastes are converted into L-Phe. Given that we have verified the stoichiometry of L-Phe conversion into styrene, we can estimate the production of 8.5 tons of styrene in a 2G biorefinery capable of processing 1000 tons of feedstock daily, equivalent to 3100 tons of styrene annually. Therefore, for a more efficient biofactory effort to increase the intracellular production of L-Phe, the starting chemical for styrene biosynthesis is needed.

To sum up our results demonstrate the success of the “wholesale” approach for designing enzymes able to convert *trans*-cinnamic acid into styrene and capable of functioning actively in novel hosts. These findings open up new avenues for the design of new-to-nature pathways for the synthesis of high-value added chemicals, marking a significant stride towards the sustainable bioproduction of chemicals.

### Supplementary Information

**Additional file 1: Figure S1.** Phylogenetic relationships of FDC1 from *Saccharomyces cerevisiae* with homologous sequences from different organisms exhibiting > 50% identity. Colors of the branches represent levels of significance obtained in the bootstrapping analysis (1000 replicates).

**Additional file 2: Figure S2.** Multialignment of FDC1 from *S. cerevisiae* and FDC from different organisms exhibiting > 60% identity. The consensus sequence derived from the multialignment was used to design the PSC1 protein.

**Additional file 3: Figure S3.** Multialignment of FDC1 from *S. cerevisiae* and FDC from different organisms exhibiting > 50% identity. The consensus sequence derived from the multialignment was used to design the PSD1 protein.

**Additional file 4: Figure S4.** Identification of styrene produced by *P. putida* bearing the PSC1 gene. Head to tail comparison of the standard mass spectra showing the relative abundance of the mass-to-charge ratio of styrene from the NIST17 library (lower) with that of the dominant metabolite peak obtained in *P. putida* CM12-5 pPSC1 culture from glucose and 0.25 mM *trans-*cinnamic acid added to the medium (upper).

**Additional file 5: Table S1.** Similar proteins (> 50% identity) to FDC1 from *Saccharomyces cerevisiae* obtained using BLAST.

**Additional file 6: Table S2.** Symbols present in the consensus sequences derived from multi-alignment of FDC homologous proteins and amino acids to replace them at the indicated position.

**Additional file 7: Table S3.** DNA and protein sequences of PSC1.

**Additional file 8: Table S4.** DNA and protein sequences of PSD1.

## Data Availability

All data reported in this MS are available in the main body or as Additional file.

## Abbreviations

Rif^R^: Rifampicin-resistant
Gm^R^: Gentamycin-resistant
Km^R^: Kanamycin-resistant
Tol^R^: Toluene tolerant
L-Phe: L-phenylalanine
*t*CA: *trans*-Cinnamic acid
*ori*pBBR1: Origin of replication pBBR1
*xylS*-Pm: XylS-Pm regulator/promoter system
FDC: Ferulic acid decarboxylase
PAL: Phenylalanine ammonia lyase

## References

[1] de Meester C, Poncelet F, Roberfroid M, Rondelet J, Mercier M (1977). Mutagenicity of styrene and styrene oxide. Mutat Res Fundam Mol Mech Mutagen.

[2] IARC (2019). Styrene, styrene-7,8-oxide, and quinoline.

[3] McKenna R, Nielsen DR (2011). Styrene biosynthesis from glucose by engineered *E*. *coli*. Metab Eng.

[4] ChemAnalyst. Styrene market size, share, industry analysis and forecast. 2030. 2023. https://www.chemanalyst.com/industry-report/styrene-market-650. Accessed 20 Dec 2020.

[5] Wu CY, Kobylinski TP, Bozik JE (1981). Preparation of styrene from ethylbenzene.

[6] Worrell E, Phylipsen D, Einstein D, Martin N (2000). Energy use and energy intensity of the U.S. chemical industry.

[7] Gilbert HJ, Tully M (1982). Synthesis and degradation of phenylalanine ammonia-lyase of *Rhodosporidium toruloides*. J Bacteriol.

[8] Moffitt MC, Louie GV, Bowman ME, Pence J, Noel JP, Moore BS (2007). Discovery of two cyanobacterial phenylalanine ammonia lyases: kinetic and structural characterization. Biochemistry.

[9] Qi WW, Vannelli T, Breinig S, Ben-Bassat A, Gatenby AA, Haynie SL (2007). Functional expression of prokaryotic and eukaryotic genes in *Escherichia coli* for conversion of glucose to *p*-hydroxystyrene. Metab Eng.

[10] Vannelli T, Xue Z, Breinig S, Qi WW, Sariaslani FS (2007). Functional expression in *Escherichia coli* of the tyrosine-inducible tyrosine ammonia-lyase enzyme from yeast *Trichosporon cutaneum* for production of *p*-hydroxycinnamic acid. Enzyme Microb Technol.

[11] Xiang L, Moore BS (2005). Biochemical characterization of a prokaryotic phenylalanine ammonia lyase. J Bacteriol.

[12] Young MR, Neisht AC (1966). Properties of the ammonia-lyases deaminating phenylalanine and related compounds in *Triticum aestivum* and *Pteridium aquilinum*. Phytochemistry.

[13] Mukai N, Masaki K, Fujii T, Kawamukai M, Iefuji H (2009). PAD1 and FDC1 are essential for the decarboxylation of phenylacrylic acids in *Saccharomyces cerevisiae*. J Biosci Bioeng.

[14] McKenna R, Thompson B, Pugh S, Nielsen DR (2014). Rational and combinatorial approaches to engineering styrene production by *Saccharomyces cerevisiae*. Microb Cell Fact.

[15] Weber FJ, de Bont JAM (1996). Adaptation mechanisms of microorganisms to the toxic effects of organic solvents on membranes. Biochim Biophys Acta.

[16] García-Franco A, Godoy P, Duque E, Ramos JL (2023). Insights into the susceptibility of *Pseudomonas putida* to industrially relevant aromatic hydrocarbons that it can synthesize from sugars. Microb Cell Fact.

[17] Horinouchi T, Tamaoka K, Furusawa C, Ono N, Suzuki S, Hirasawa T (2010). Transcriptome analysis of parallel-evolved *Escherichia coli* strains under ethanol stress. BMC Genomics..

[18] Segura A, Godoy P, van Dillewijn P, Hurtado A, Arroyo N, Santacruz S (2005). Proteomic analysis reveals the participation of energy-and stress-related proteins in the response of *Pseudomonas putida* DOT-T1E to toluene. J Bacteriol.

[19] Ramos JL, Duque E, Godoy P, Segura A (1998). Efflux pumps involved in toluene tolerance in *Pseudomonas putida* DOT- T1E. J Bacteriol.

[20] Rojas A, Segura A, Guazzaroni E, Terán W, Hurtado A, Gallegos T (2003). In vivo and in vitro evidence that TtgV Is the specific regulator of the TtgGHI multidrug and solvent efflux pump of *Pseudomonas putida*. J Bacteriol.

[21] Bernal P, Segura A, Ramos JL (2007). Compensatory role of the *cis-trans*-isomerase and cardiolipin synthase in the membrane fluidity of *Pseudomonas putida* DOT-T1E. Environ Microbiol.

[22] Junker F, Ramos JL (1999). Involvement of the *cis/trans* isomerase Cti in solvent resistance of *Pseudomonas putida* DOT-T1E. J Bacteriol.

[23] Ramos JL, Cuenca MS, Molina-Santiago C, Segura A, Duque E, Gómez-García MR (2015). Mechanisms of solvent resistance mediated by interplay of cellular factors in *Pseudomonas putida*. FEMS Microbiol Rev.

[24] Udaondo Z, Molina L, Segura A, Duque E, Ramos JL (2016). Analysis of the core genome and pangenome of *Pseudomonas putida*. Environ Microbiol.

[25] Godoy P, García-Franco A, Recio MI, Ramos JL, Duque E (2021). Synthesis of aromatic amino acids from 2G lignocellulosic substrates. Microb Biotechnol.

[26] Molina-Santiago C, Cordero BF, Daddaoua A, Udaondo Z, Manzano J, Valdivia M (2016). *Pseudomonas putida* as a platform for the synthesis of aromatic compounds. Microbiology (N Y).

[27] Sahdev S, Khattar SK, Saini KS (2008). Production of active eukaryotic proteins through bacterial expression systems: a review of the existing biotechnology strategies. Mol Cell Biochem.

[28] Khow O, Suntrarachun S (2012). Strategies for production of active eukaryotic proteins in bacterial expression system. Asian Pac J Trop Biomed.

[29] Sternke M, Tripp KW, Barrick D (2019). Consensus sequence design as a general strategy to create hyperstable, biologically active proteins. Proc Natl Acad Sci USA.

[30] Sternke M, Tripp KW, Barrick D (2020). The use of consensus sequence information to engineer stability and activity in proteins. Methods Enzymol.

[31] Abril MA, Michan C, Timmis KN, Ramos JL (1989). Regulator and enzyme specificities of the TOL plasmid-encoded upper pathway for degradation of aromatic hydrocarbons and expansion of the substrate range of the pathway. J Bacteriol.

[32] Martínez-García E, Nikel PI, Aparicio T, De Lorenzo V (2014). Pseudomonas 2.0: genetic upgrading of *P*. *putida* KT2440 as an enhanced host for heterologous gene expression. Microb Cell Fact..

[33] Martínez-García E, Aparicio T, Goñi-Moreno A, Fraile S, De Lorenzo V (2015). SEVA 2.0: an update of the Standard European Vector Architecture for de-/re-construction of bacterial functionalities. Nucleic Acids Res..

[34] Altschul SF, Madden TL, Schäffer AA, Zhang J, Zhang Z, Miller W (1997). Gapped BLAST and PSI-BLAST: a new generation of protein database search programs. Nucleic Acid Res.

[35] Boutet E, Lieberherr D, Tognolli M, Schneider M, Bairoch A (2007). UniProtKB/Swiss-Prot. Methods Mol Biol.

[36] Silva-Rocha R, Martínez-García E, Calles BN, Chavarría M, Arce-Rodríguez A, De Las HA (2013). The Standard European Vector Architecture (SEVA): a coherent platform for the analysis and deployment of complex prokaryotic phenotypes. Nucleic Acids Res.

[37] Ramos JL, Wasserfallen A, Rose K, Timmis KN (1987). Redesigning metabolic routes: manipulation of TOL plasmid pathway for catabolism of alkylbenzoates. Science.

[38] Nguyen L-T, Schmidt HA, von Haeseler A, Minh BQ (2015). IQ-TREE: a fast and effective stochastic algorithm for estimating maximum-likelihood phylogenies. Mol Biol Evol.

[39] Hoang DT, Chernomor O, von Haeseler A, Minh BQ, Vinh LS (2018). UFBoot2: improving the ultrafast bootstrap approximation. Mol Biol Evol.

[40] Kalyaanamoorthy S, Minh BQ, Wong TK, von Haeseler A, Jermiin LS (2017). ModelFinder: fast model selection for accurate phylogenetic estimates Europe PMC funders group. Nat Methods.

[41] Michaud JM, Madani A, Fraser JS (2022). A language model beats alphafold2 on orphans. Nat Biotechnol.

[42] Fernandez-Escamilla AM, Rousseau F, Schymkowitz J, Serrano L (2004). Prediction of sequence-dependent and mutational effects on the aggregation of peptides and proteins. Nat Biotechnol.

[43] Yang J, Zhang Y (2015). I-TASSER server: new development for protein structure and function predictions. Nucleic Acids Res.

[44] Moore ERB, Tindall BJ, Martins Dos Santos VAP, Pieper DH, Ramos JL, Palleroni NJ, Dworkin M, Falkow S, Rosenberg E, Schleifer KH, Stackebrandt E (2006). Nonmedical: *Pseudomonas*. The Prokaryotes.

[45] Ramos JL, Duque E, Huertas MJ, Haïdour A (1995). Isolation and expansion of the catabolic potential of a *Pseudomonas putida* strain able to grow in the presence of high concentrations of aromatic hydrocarbons. J Bacteriol.

[46] Bird LJ, Mickol RL, Eddie BJ, Thakur M, Yates MD, Glaven SM (2023). *Marinobacter*: a case study in bioelectrochemical chassis evaluation. Microb Biotechnol.

[47] Danchin A (2022). *In vivo*, *in vitro* and *in silico*: an open space for the development of microbe-based applications of synthetic biology. Microb Biotechnol.

[48] Bailey SS, Payne KAP, Fisher K, Marshall SA, Cliff MJ, Spiess R (2018). The role of conserved residues in Fdc decarboxylase in prenylated flavin mononucleotide oxidative maturation, cofactor isomerization, and catalysis. J Biol Chem.

[49] Duță H, Filip A, Nagy LC, Nagy EZA, Tőtős R, Bencze LC (2022). Toolbox for the structure-guided evolution of ferulic acid decarboxylase (FDC). Sci Rep.

[50] Lee K, Bang HB, Lee YH, Jeong KJ (2019). Enhanced production of styrene by engineered *Escherichia coli* and in situ product recovery (ISPR) with an organic solvent. Microb Cell Fact.

[51] Grubbe WS, Rasor BJ, Krüger A, Jewett MC, Karim AS (2020). Cell-free styrene biosynthesis at high titers. Metab Eng.

[52] Zhan Y, Shi J, Xiao Y, Zhou F, Wang H, Xu H (2022). Multilevel metabolic engineering of *Bacillus licheniformis* for de novo biosynthesis of 2-phenylethanol. Metab Eng.

[53] Liu C, Men X, Chen H, Li M, Ding Z, Chen G (2018). A systematic optimization of styrene biosynthesis in *Escherichia coli* BL21(DE3). Biotechnol Biofuels.

[54] McKenna R, Moya L, McDaniel M, Nielsen DR (2015). Comparing in situ removal strategies for improving styrene bioproduction. Bioprocess Biosyst Eng.

[55] Liang L, Liu R, Foster KEO, Choudhury A, Cook S, Cameron JC (2020). Genome engineering of *E. coli* for improved styrene production. Metab Eng..

