# Supplementary figures and images for: Engineering styrene biosynthesis: designing a functional trans-cinnamic acid decarboxylase in Pseudomonas
Source: Microb Cell Fact. 2024 Feb 28;23:69. doi: 10.1186/s12934-024-02341-0 (PMC10903017; doi:10.1186/s12934-024-02341-0)

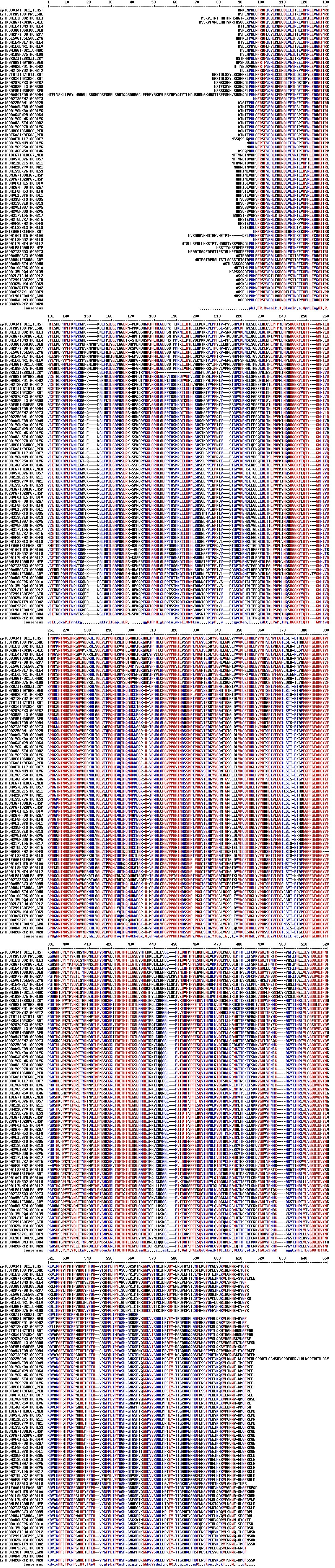

Supplement: Supplementary file 3 — Additional file 3: Figure S3. Multialignment of FDC1 from S. cerevisiae and FDC from different organisms exhibiting > 50% identity. The consensus sequence derived from the multialignment was used to design the PSD1 protein. [file 12934_2024_2341_MOESM3_ESM.tiff]

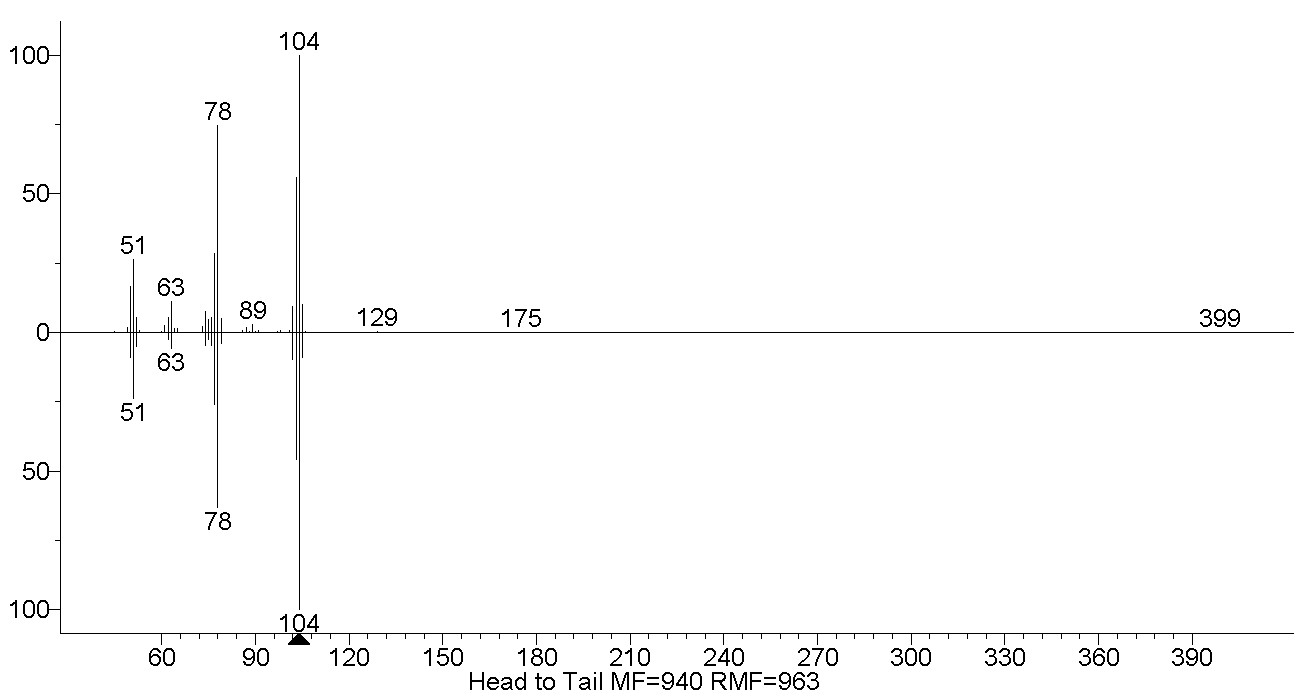

Supplement: Supplementary file 4 — Additional file 4: Figure S4. Identification of styrene produced by P. putida bearing the PSC1 gene. Head to tail comparison of the standard mass spectra showing the relative abundance of the mass-to-charge ratio of styrene from the NIST17 library (lower) with that of the dominant metabolite peak obtained in P. putida CM12-5 pPSC1 culture from glucose and 0.25 mM trans-cinnamic acid added to the medium (upper). [file 12934_2024_2341_MOESM4_ESM.tiff]
