# Supplementary material for: Engineering styrene biosynthesis: designing a functional trans-cinnamic acid decarboxylase in Pseudomonas
Source: Microb Cell Fact. 2024 Feb 28;23:69. doi: 10.1186/s12934-024-02341-0 (PMC10903017; doi:10.1186/s12934-024-02341-0)
Supplement: Supplementary file 5 — Additional file 5: Table S1. Similar proteins (> 50% identity) to FDC1 from Saccharomyces cerevisiae obtained using BLAST. [file 12934_2024_2341_MOESM5_ESM.docx]

| Protein and species | Length^a^ | Identity percentage (%)^b^ | UniProtKB accession number |
| --- | --- | --- | --- |
| FDC1 *Saccharomyces kudriavzevii* | 503 | 88.9 | J8TRN5 |
| FDC1 *Wickerhamomyces anomalus* | 518 | 68.2 | A0A1E3P002 |
| FDC1 *Wickerhamomyces ciferrii* | 523 | 63.7 | K0KNG7 |
| FDC1 *[Candida] pseudohaemulonis* | 512 | 61.2 | A0A2P7YF98 |
| FDC1 *Debaryomyces hansenii* | 513 | 61.1 | Q6BJQ8 |
| FDC1 *Candida dubliniensis* | 513 | 60.6 | B9WJ66 |
| FDC1 *[Candida] arabinofermentans* | 501 | 60.0 | A0A1E4T849 |
| FDC1 *Candida albicans* | 513 | 59.8 | A0A1D8PQ75 |
| FDC1 *Zygosaccharomyces rouxii* | 511 | 59.6 | C5E506 |
| FDC1 *Hyphopichia burtonii* | 510 | 58.6 | A0A1E4RBI7 |
| FDC1 *[Candida] intermedia* | 511 | 56.7 | A0A1L0D4X1 |
| FDC1 *Cryptococcus gattii serotype B (strain WM276 / ATCC MYA-4071) (Filobasidiella gattii) (Cryptococcus bacillisporus)* | 435 | 54.1 | E6R9Z1 |
| FDC1 *Capronia coronata* | 498 | 53.8 | W9YNA8 |
| 3-octaprenyl-4-hydroxybenzoate carboxy-lyase *Metarhizium majus (strain ARSEF 297)* | 411 | 53.1 | A0A0B4HJM3 |
| FDC1 *Cryptococcus gattii serotype B* (*strain R265) (Filobasidiella gattii)* (Cryptococcus bacillisporus) | 501 | 52.5 | A0A095C6V3 |
| FDC1 *Neonectria ditissima* | 507 | 52.2 | A0A0N8H5Z4 |
| Multifunctional fusion protein *Talaromyces atroroseus* | 1348 | 52.2 | A0A225AAN1 |
| FDC1 *Corynespora cassiicola Philippines* | 497 | 51.8 | A0A2T2NYU2 |
| FDC1 *Phialophora americana* | 499 | 51.8 | A0A0D2DPQ1 |
| FDC1 *Botryotinia fuckeliana (strain BcDW1)* | 513 | 51.8 | M7THT1 |
| FDC1 *Botryotinia fuckeliana (strain T4)* | 513 | 51.6 | G2XWX0 |
| FDC1 *Neosartorya fischeri* | 505 | 51.4 | A1DCG7 |
| FDC1 *Fusarium sp. AF-8* | 406 | 51.4 | A0A428NRY2 |
| FDC1 *Cryptococcus gattii serotype B (strain WM276 / ATCC MYA-4071) (Filobasidiella gattii) (Cryptococcus bacillisporus)* | 523 | 51.4 | E6RA84 |
| FDC1 *Rutstroemia sp. NJR-2017a WRK4* | 511 | 51.2 | A0A2S7QZX3 |
| FDC1 *Aspergillus turcosus* | 504 | 51.1 | A0A421CVP0 |
| FDC1 *Pseudogymnoascus sp. VKM F-4520 (FW-2644)* | 589 | 51.0 | A0A094IED9 |
| FDC1 *Colletotrichum fioriniae PJ7* | 503 | 51.0 | A0A010QFR6 |
| FDC1 *Penicillium nordicum* | 500 | 51.0 | A0A0M9WF89 |
| FDC1 *Penicillium polonicum* | 500 | 50.9 | A0A1V6NK80 |
| *FDC1 Penicillium digitatum (strain PHI26 / CECT 20796) (Green mold)* | 499 | 50.9 | K9FG02 |
| FDC1 *Aspergillus lentulus* | 505 | 50.9 | A0A0S7DJV6 |
| FDC1 *Aspergillus oryzae* | 503 | 50.9 | A0A1S9DK76 |
| FDC1 *Aspergillus flavus* | 503 | 50.9 | B8NJ67 |
| FDC1 *Aspergillus oryzae* | 503 | 50.9 | Q2UP67 |
| FDC1 *Aspergillus parasiticus* | 503 | 50.9 | A0A0F0IHE5 |
| FDC1 *Aspergillus arachidicola* | 503 | 50.9 | A0A2G7FFD8 |
| FDC1 *Hypocrea atroviridis* | 512 | 50.8 | G9NLP8 |
| FDC1 *Grosmannia clavigera* | 500 | 50.8 | F0XL98 |
| FDC1 *Trichoderma asperellum CBS 433.97* | 525 | 50.8 | A0A2T3Z5Q3 |
| FDC1 *Aspergillus homomorphus* | 503 | 50.8 | A0A395HXT9 |
| FDC1 *Penicillium camemberti FM 013* | 500 | 50.7 | A0A0G4P429 |
| FDC1 *Aspergillus novofumigatus IBT 16806* | 505 | 50.7 | A0A2I1BZC5 |
| FDC1 *Aspergillus bombycis* | 503 | 50.7 | A0A1F8AA53 |
| FDC1 *Aspergillus ochraceoroseus IBT 24754* | 502 | 50.7 | A0A2T5LVK7 |
| FDC1 *Fusarium venenatum* | 503 | 50.6 | A0A2L2TCJ4 |
| FDC1 *Aspergillus uvarum CBS 121591* | 503 | 50.6 | A0A319C3E8 |
| FDC1 *Penicillium expansum* | 500 | 50.5 | A0A0A2J5F4 |
| FDC1 *Penicillium brasilianum* | 503 | 50.5 | A0A0F7U117 |
| FDC1 *Aspergillus sclerotioniger CBS 115572* | 505 | 50.5 | A0A317V105 |
| FDC1 *Sphaerulina musiva* | 508 | 50.5 | M3DF95 |
| FDC1 *Coleophoma cylindrospora* | 513 | 50.4 | A0A3D8RLL3 |
| FDC1 *Penicillium decumbens* | 497 | 50.4 | A0A1V6NNH9 |
| FDC1 *Penicillium solitum* | 500 | 50.3 | A0A1V6RL46 |
| FDC1 *Aspergillus nomius NRRL 131317* | 503 | 50.3 | A0A0L1J9Y6 |
| FDC1 *Gibberella fujikuroi* | 503 | 50.2 | S0E299 |
| FDC1 *Gibberella intermedia (Bulb rot disease fungus) (Fusarium proliferatum)* | 503 | 50.2 | A0A365NJK4 |
| FDC1 *Fusarium kuroshium* | 503 | 50.2 | A0A3M2RIT9 |
| FDC1 *Aspergillus violaceofuscus* | 503 | 50.2 | A0A2V5HJD9 |
| FDC1 *Amorphotheca resinae ATCC 22711* | 496 | 50.2 | A0A2T3AZN7 |
| FDC1 *Aspergillus indologenus CBS 114.80* | 503 | 50.2 | A0A2V5I997 |
| FDC1 *Penicillium flavigenum* | 500 | 50.1 | A0A1V6SP78 |
| FDC1 *Penicillium rubens* | 500 | 50.1 | B6HRC8 |
| FDC1 *Trichoderma harzianum* | 499 | 50.1 | A0A0F9Z7X1 |
| FDC1 *Aspergillus rambellii* | 497 | 50.1 | A0A0F8UFA2 |
| FDC1 *Aspergillus luchuensis* | 500 | 50.1 | A0A146FW50 |
| FDC1 *Aspergillus niger* | 517 | 50.0 | A0A100IUI5 |
| FDC1 *Aspergillus tubingensis* | 493 | 50.0 | A0A1L9N5Q2 |
| FDC1 *Penicillium steckii* | 495 | 50.0 | A0A1V6SR50 |
| FDC1 *Botryosphaeria parva* | 495 | 50.0 | R1EM06 |
| FDC1 *Colletotrichum salicis* | 505 | 50.0 | A0A135URQ4 |
| FDC1 *Penicilliopsis zonata CBS 506.65* | 498 | 50.0 | A0A1L9S913 |
| FDC1 *Phialocephala subalpina* | 528 | 50.0 | A0A1L7WNI4 |

^a^ Protein length (number of amino acids).

^b^ Percentage of residues identical to those of the enzyme *trans*-cinnamic acid decarboxylase from *Saccharomyces cerevisiae* (*Ferulic acid decarboxylase*, FDC1).
