# Supplementary material for: Engineering styrene biosynthesis: designing a functional trans-cinnamic acid decarboxylase in Pseudomonas
Source: Microb Cell Fact. 2024 Feb 28;23:69. doi: 10.1186/s12934-024-02341-0 (PMC10903017; doi:10.1186/s12934-024-02341-0)
Supplement: Supplementary file 6 — Additional file 6: Table S2. Symbols present in the consensus sequences derived from multi-alignment of FDC homologous proteins and amino acids to replace them at the indicated position. [file 12934_2024_2341_MOESM6_ESM.docx]

| Symbol | Amino acids |
| --- | --- |
| ! | Isoleucine (I) o Valine (V) |
| $ | Leucine (L) o Methionine (M) |
| % | Phenylalanine (F) o Tyrosine (Y) |
| # | Asparagine (N), Aspartic Acid (D), Glutamine (Q), Glutamic Acid (E) |
