# Supplementary material for: Engineering styrene biosynthesis: designing a functional trans-cinnamic acid decarboxylase in Pseudomonas
Source: Microb Cell Fact. 2024 Feb 28;23:69. doi: 10.1186/s12934-024-02341-0 (PMC10903017; doi:10.1186/s12934-024-02341-0)
Supplement: Supplementary file 8 — Additional file 8: Table S4. DNA and protein sequences of PSD1. [file 12934_2024_2341_MOESM8_ESM.docx]

| **DNA sequence of *psd1*** |
| --- |
| ATGCCGCACCTGTTCCGCTTCGTGGAAGCCCTGAAAGATGACCTGGTGGAAATCAACGAAGATCCGAACCTGGAAGCCGGTGCCATCCGTGTGTGCGAGACCGACAAAGCCCCGCTGTTCAACAACCTGAAGGGCGGCCTGTTCCGCATCCTGGGTGCCCCGAGCCTGCGTTATGGTCGCCTGGCCCGCCATCTGGGCCTGCCGCCGACCGCCATGAAGGAAATCCTGGATAAAATGCTGAGCGCGGCCGCCGCCGCCCCGATCCCGCCGGCCGCCGTGGCCACCGGCCCGTGCAAGGAGAACGCCCTGGCGGCGGCCGCCATCGATCTGACCGCCCTGCCGGCCCCGGCCCTGCACCAGGCCGACGGTGGCAAATACATCCAGACCTATGGCATGCATATCGTGCAGAGCCCGGATGGCAAGTGGACCAACTGGAGCATCGCCCGTGCCATGGTGGCCGACGCCAACCACCTGGCCGGCCTGGTGATCGAACCGCAGCACATCTGGCAGATCCATCAGATGTGGAAGAAAGAGGGCAAAGATGTGCCGTGGGCCCTGGCCTTCGGTGTGCCGCCGGCGGCCATCGCCAGCAGCATGCCGATCCCGGATGGTGTGAGCGAAGCCGCCTATGTGGGTGCCGCCGCCGGTGCCGCCCTGGCCGTGGTGAAGTGCGATACCAACGACCTGGCCGTGCCGGCCGCCAGCGAAATCGTGTTCGAGGGCACCCTGAGCATCACCGAAACCGCCCCGGAGGGTCCGTTCGGTGAAATGCACGGCTATGTGTTCCCGGGTGATGCCCATGCCGCCCCGGCCTACGCCGTGAACGCCATCACCTATCGTGCCGCCGCCATCCTGCCCGTGAGCAACTGCGGTCGTCTGACCGACGAAACCCACACCATGATCGGCGCCCTGGCCGCCGCCGAGATCGCCGCCGCCTGCGCGGCCGCCGGTCTGCCGATCGCCGATGCCTTCGCCCCGTTCGAAAGCCAGGTGACCTGGGTGGCCCTGAAGGTGGACACCGCCAAACTGCGTGCCATGAAGACCACCCCGGCCGAGTTCGCCAAAGCCGTGGGTGACGCCGTGTTCAACCACAAGGCCGGCTACACCATCCATCGCCTGGTGCTGGTGGGCGATGACATCGATGTGTATAACGCCAAAGATGTGTGGGCCTTCACCCGTTGCCGTCCGGATGAGTTCTTCGAGGACGTGGGCTTCCCGCTGATCCCGTATATGAGCCATGGTGGCCCGGGTGGCAAGGTTGTGAGCGATGCCCTGCCGGAATACACCGGTTGGGCCGACTTCAGCTATCCGGAGAAGAAAGTGTGGGGCTTCTAA |
| **Protein sequence of PSD1** |
| MPHLFRFVEALKDDLVEINEDPNLEAGAIRVCETDKAPLFNNLKGGLFRILGAPSLRYGRLARHLGLPPTAMKEILDKMLSAAAAAPIPPAAVATGPCKENALAAAAIDLTALPAPALHQADGGKYIQTYGMHIVQSPDGKWTNWSIARAMVADANHLAGLVIEPQHIWQIHQMWKKEGKDVPWALAFGVPPAAIASSMPIPDGVSEAAYVGAAAGAALAVVKCDTNDLAVPAASEIVFEGTLSITETAPEGPFGEMHGYVFPGDAHAAPAYAVNAITYRAAAILPVSNCGRLTDETHTMIGALAAAEIAAACAAAGLPIADAFAPFESQVTWVALKVDTAKLRAMKTTPAEFAKAVGDAVFNHKAGYTIHRLVLVGDDIDVYNAKDVWAFTRCRPDEFFEDVGFPLIPYMSHGGPGGKVVSDALPEYTGWADFSYPEKKVWGF |
